# Robotic-assisted nephrectomy with level II IVC thrombectomy using Rummel Tourniquets

**DOI:** 10.1590/S1677-5538.IBJU.2021.0393

**Published:** 2021-09-10

**Affiliations:** 1 Midwestern University Arizona College of Osteopathic Medicine Glendale AZ USA Arizona College of Osteopathic Medicine, Midwestern University, Glendale, AZ, USA; 2 University of Michigan Department of Urology Wyoming MI USA Department of Urology, Metro Health, University of Michigan, Wyoming, MI, USA

## Abstract

**Background:**

Inferior vena cava (IVC) invasion from renal cell carcinoma (RCC) occurs at a rate of 4-10% (1). IVC thrombectomy (IVC-TE) can be an open procedure because of the need for handling of the IVC (2). The first reported series of robotic management of IVC-TE started in 2011 for the management of Level I - II thrombi with subsequent case reports in recent years (2-5).

**Materials and Methods:**

The following is a patient in his 50’s with no significant medical history. Magnetic resonance imaging and IR venogram were performed preoperatively. The tumor was clinical stage T3b with a 4.3cm inferior vena cava thrombus. The patient underwent robotic assisted nephrectomy and IVC-TE. Rummel tourniquets were used for the contralateral kidney and the IVC. The tourniquets were created using vessel loops, a 24 French foley catheter and hem-o-lock clips.

**Results:**

The patient tolerated the surgical procedure well with no intraoperative complications. Total surgical time was 274 min with 200 minutes of console time and 22 minutes of IVC occlusion. Total blood loss in the surgery was 850cc. The patient was discharged from the hospital on post-operative day 3 without any complications. The final pathology of the specimen was pT3b clear cell renal cell carcinoma Fuhrman grade 2. The patient followed up post-operatively at both four months and six months without disease recurrence. The patient continues annual follow-up with no recurrence.

**Conclusions:**

Surgeon experience is a key factor in radical nephrectomy with thrombectomy as patients have a reported 50-65% survival rate after IVC-TE (4).

## AUTHOR DISCLOSURE STATEMENT

Authors have received and archived patient consent for video recording/publication in advance of video recording procedure.

## SOURCE OF WORK

All work is original from TM and Metro Health - University of Michigan.

## ABBREVIATIONS

IVC: Inferior vena cava
RCC: renal cell carcinoma
IVC-TE: IVC thrombectomy
IR: interventional radiology
